# Efficacy and safety of switching to bilastine, an H1-antihistamine, in patients with refractory chronic spontaneous urticaria (H1-SWITCH): a multicenter, open-label, randomized, parallel-group comparative study

**DOI:** 10.3389/fimmu.2024.1441478

**Published:** 2024-09-16

**Authors:** Atsushi Fukunaga, Yasumasa Kakei, Sae Murakami, Yuji Kan, Koji Masuda, Masatoshi Jinnin, Ken Washio, Hiroo Amano, Tohru Nagano, Akihisa Yamamoto, Toshihiro Otsuka, Shunsuke Takahagi, Motoi Takenaka, Naoko Ishiguro, Koremasa Hayama, Naoko Inomata, Yukinobu Nakagawa, Akiko Sugiyama, Michihiro Hide

**Affiliations:** ^1^ Department of Dermatology, Division of Medicine for Function and Morphology of Sensory Organs, Faculty of Medicine, Osaka Medical and Pharmaceutical University, Takatsuki, Japan; ^2^ Department of Dermatology, Kobe University Graduate School of Medicine, Kobe, Japan; ^3^ Department of Oral and Maxillofacial Surgery, Kobe University Graduate School of Medicine, Kobe, Japan; ^4^ Clinical and Translational Research Center, Kobe University Hospital, Kobe, Japan; ^5^ Department of Dermatology, Sapporo Medical University School of Medicine, Sapporo, Japan; ^6^ Department of Dermatology, Kyoto Prefectural University of Medicine Graduate School of Medical Science, Kyoto, Japan; ^7^ Department of Dermatology, Wakayama Medical University, Wakayama, Japan; ^8^ Department of Dermatology, Kobe City Nishi-Kobe Medical Center, Kobe, Japan; ^9^ Department of Dermatology, Iwate Medical University School of Medicine, Shiwa-gun, Japan; ^10^ Department of Dermatology, Kobe City Medical Center General Hospital, Kobe, Japan; ^11^ Department of Dermatology, Takarazuka City Hospital, Takarazuka, Japan; ^12^ Department of Dermatology, School of Biomedical and Health Sciences, Hiroshima University, Hiroshima, Japan; ^13^ Department of Dermatology, Nagasaki University School of Biomedical Sciences, Nagasaki, Japan; ^14^ Department of Dermatology, Tokyo Women’s Medical University, Shinjuku-ku, Japan; ^15^ Division of Cutaneous Science, Department of Dermatology, Nihon University School of Medicine, Tokyo, Japan; ^16^ Department of Environmental Immuno-Dermatology, Yokohama City University School of Medicine, Yokohama, Japan; ^17^ Department of Dermatology, Course of Integrated Medicine, Graduate School of Medicine, Osaka University, Suita, Japan; ^18^ Department of Allergology, National Hospital Organization (NHO), Fukuoka National Hospital, Fukuoka, Japan; ^19^ Department of Dermatology, Hiroshima City Hiroshima Citizens Hospital, Hiroshima, Japan

**Keywords:** chronic spontaneous urticaria, histamine H1 antagonists, Japan, sleepiness, quality of life, switching to bilastine

## Abstract

**Background:**

For treating patients with refractory chronic spontaneous urticaria (CSU) resistant to standard doses of 2^nd^ generation H1-antihistamines (H1AH) the International and Japanese guidelines recommend increasing H1AH dose. The latter also recommends switching to a different H1AH. This study explored if the efficacy of the standard dose of bilastine 20 mg is non-inferior to that of double-dose of H1AH in patients with refractory CSU.

**Methods:**

This phase IV, multicenter, open-label, randomized, parallel-group trial evaluated the efficacy and safety of switching treatment to bilastine compared to treatment with a 2-fold dose of H1AH in patients with CSU refractory to standard dose H1AH. The primary endpoint was the mean total symptom score (TSS) at Day 5-7 after the start of administration.

**Results:**

Treatment efficacy and safety were evaluated in 128 patients (bilastine, n=64; 2-fold dose of H1AH, n=64). The mean TSS at Day 5-7 after the start of administration was smaller than the non-inferiority margin of 0.8, demonstrating non-inferiority of the bilastine switching group to the double-dose H1AH group (0.17 (95% CI -0.32, 0.67)). No difference in Japanese version of Epworth Sleepiness Scale (JESS), DLQI, and urticaria activity score over 7 consecutive days (UAS7) was observed between the two groups. There were no serious adverse events in either group. H1AH-related adverse events occurred in 5 subjects (8 cases) and 2 subjects (3 cases) in the double-dose H1AH and bilastine groups, respectively.

**Conclusions:**

Switching treatment to bilastine demonstrated non-inferiority to a double-dose of H1AH in terms of efficacy in patients with CSU refractory to standard dose H1AH with a favorable safety profile.

**Clinical trial registration:**

https://jrct.niph.go.jp/latest-detail/jRCTs051180105, identifier jRCTs051180105.

## 1 Introduction

Chronic urticaria (CU) is defined as the occurrence of wheals, angioedema, or both for more than 6 weeks. The prevalence of CU reportedly differs between Asian and Western populations (1). Chronic spontaneous urticaria (CSU) is a type of CU that occurs without obvious triggers among CU. CSU is a highly prevalent skin disease affecting up to 1% of the general population, not only negatively affects the quality of life (QoL) and health of the patients but also accounts for a considerable socio-economic burden (2, 3). In comparison to chronic inducible urticaria, where symptoms are induced by temperature, solar, delayed pressure, aquagenic, cholinergic, dermography, and/or contact, is another form of CU, CSU is the more common form of CU. In Asia, the prevalence of CU is increasing, with CSU accounting for about two-thirds of all CU cases (4).

The complex etiology of CSU involves crosslinking of immunoglobulin E (IgE) bound to the high affinity IgE receptors on the surface of cutaneous mast cells or basophils followed by the release of pro-inflammatory mediators such as histamine, platelet-activating factor, and cytokines. The release of these mediators induces sensory nerve activation, vasodilatation, plasma extravasation, and cell recruitment to urticarial lesions (5). Two autoimmune endotypes (aiCSU), either Type I or Type IIb, have been associated with the activation of skin mast cells (6).

The symptoms of CSU are primarily mediated by the actions of histamine on H1-receptors located on endothelial cells, resulting in wheal formation and acting on sensory nerves, leading to neurogenic pruritus (6). In the majority of cases, this long-lasting disorder can persist for 2–5 years, while 20% of patients remain affected for more than five years (7). According to the international guideline, the treatment goal for CSU is complete control of urticaria symptoms and normalization of quality of life (8).

Continuous treatment with H1-antihistamine (H1AH) is considered as the conventional treatment of CSU, but the complete absence of symptoms is achieved only in less than 50% of these patients (9). H1AH are classified into tricyclic and piperazine/piperidine based on their structural formula. In contrast to the 1^st^ generation H1AH, non-sedating 2^nd^ generation antihistamines (sgAH) have a better safety profile, even when taken in higher doses, and thus are increasingly being considered for the first-line of treatment to alleviate the symptoms of CSU (2, 8, 10). For the poor responders to standard dose sgAH, international guidelines recommend increasing the dose of sgAHs up to 4-fold and/or adding IgE blocker omalizumab on sgAH or adding cyclosporine on sgAH (8, 11–13). However, geographical regions and races play roles in defining the clinical characteristics and management of CSU underscoring the need of customizing the treatment strategy in the context of specific patient popuations (4, 14). In a real-world survey in Japan, patients with CU having an Urticaria Control Test (UCT) score of <12 demonstrated compromised QoL and impaired productivity and activity - with 64% of patients reporting uncontrolled symptoms. Moreover, only 36.1% of patients achieved adequate control of CU (15).

In contrast to the international scenario, the health insurance system in Japan limits increasing the dose of antihistamines up to two times only. Thus, for the patients who are unresponsive to the standard dose of sgAH, doubling up the standard dose, as well as either switching to other H1AH, or combining two antihistamines is recommended (3). However, increasing the doses of H1AHs elevates the risk of side effects. Even sgAHs- despite having better safety profile owing to their less brain penetration activity compared to first-generation H1AHs can lead to side effects such as drowsiness, sedation, somnolence, fatigue, and headache (16) that severely impair the productivity and QoL of patients with CSU. Therefore, finding the treatment option for optimal control of symptoms and improved productivity, especially in adolescent and middle-aged patients, without compromising the quality of life, is imperative.

The therapeutic use of bilastine, a non-sedating piperazine derivatives sgAH, is approved in 90 countries for patients with urticaria and allergic rhinitis. For adult patients, the recommended dose is 20 mg once daily. Compared to the placebo, the use of bilastine significantly improved the symptoms during the early stage (Days 1–3) of treatment (17). The use of bilastine has shown promise in terms of better symptom control at an early stage of treatment and a low incidence of side effects (18, 19). Thus, switching to bilastine can be an effective alternative for managing patients with CSU who are nonresponsive to second-generation H1AH at the standard dose.

Some retrospective studies have suggested efficacy and tolerability of bilastine in Indian patients with poor responsiveness to other sgAH (20, 21). However, no prospective and relatively large-scale study has explored the efficacy of switching to bilastine or any other specific H1AH in patients with CSU who are resistant to a certain standard dose sgAH. Furthermore, there is no previous study on CSU that has directly compared increasing the dose with switching other specific H1AH. To bridge this gap, we conducted a phase IV, investigator-initiated, multicenter, randomized, two-arm clinical trial that included Japanese patients with CSU who were resistant to treatment with standard doses of sgAHs other than bilastine.

## 2 Materials and methods

### 2.1 Study design and procedure

Our clinical trial protocol has been previously described (22) and **Supplementary Table 1** provides a summary of the study. The patients enrolled in this study met all the eligibility criteria (**Supplementary Table 2**). Briefly, this study is designed in Japanese patients with CSU who were resistant to treatment with standard doses of sgAHs other than bilastine (UCT<11) as a multicenter, open-label, randomized, parallel, comparison study.

### 2.2 Patient recruitment and randomization

We determined the sample size based on the primary endpoint. As described in Fukunaga et al. (22), based on the domestic phase III randomized controlled trial that was conducted to obtain approval to use bilastine to treat chronic urticaria (17, 18), the common standard deviation of the mean the total symptom score (TSS) of 5–7 days after bilastine administration in group B and in group A to be 1.7 and the difference between two means to be 0 were assumed. TSS is defined as the sum of the rash (synthetic; maximum 3 points per day) and itch (mean of daytime and nighttime; maximum 4 points per day) scores. Under these assumptions and a non-inferiority margin of 0.8, with one-sided significance level of 2.5% and the statistical power of 80%, the number of needed subjects based on a statistical test to confirm non-inferiority of bilastine comparing with other H1-antihistamines was estimated to be 71 per group. Considering the uncertainty and omissions that result from estimation, the sample size was set to 75 subjects per each group of H1AH double-dose group and 75 in the bilastine-switching group. However, due to the COVID-19 outbreak, patients were often unable to come to the hospital, and thus the progress in case enrollment could not be secured as originally planned. As a countermeasure, the enrollment period was extended (from 1.5 years to 3.5 years; Until November 2022) and additional sites were added (from 15 to 31 sites). Regarding the increase in the number of research facilities and the extension of the enrollment period, an ethical review was conducted at a representative institution as a specific clinical research in Japan. The final number of cases enrolled was 129 compared to the target of 150 (64 in the each group) due to the effects of the COVID-19 pandemic, which resulted in a reduction in medical visits and an increase in the number of cases using omalizumab, which was set as an exclusion criterion.

Subjects were randomly assigned to either the bilastine group or the double-dose of H1AH group at a 1:1 allocation Randomization was conducted using stratified block randomization method, with stratification based on UCT category (<8 points, or ≥8 points) (22). The block sizes were 6 and 4, each maintaining a 1:1 ratio. The principal investigator or sub-investigator sent a “Subject Enrollment Form” by Fax to the data center. The staff at the data center confirmed the subject’s eligibility and issue the “Subject Enrollment Confirmation Form” that contains the eligibility judgement result, the randomization assignment resulted from the generated random sequence, and the enrollment number. Thereafter, the form was sent to the principal investigator or sub-investigator.

### 2.3 Intervention

Patients were randomized in the following two groups:

Double dose of orally administered H1AH-group (H1AH double-dose group)- The regular dose of H1AH, that was administered orally before registration, was increased by two-fold. Oral H1AH regimen (number of oral medication per day) was not changed, which was the same as regular dose, but the dose was doubled from the night of randomization. The administration period was 7 days, and the H1AH was taken daily beginning on Day 1 (first prescription day). Medications administered twice daily was taken until the morning of Day 8. The names of H1AH drugs and the number of patients assigned to the double dose group and bilastine-switching group are listed in **Supplementary Table 4**.

Biastine switching group- The regular dose of H1AH was switched to the regimen of bilastine 20 mg, which is the approved regular dose for CSU in Japan, orally administered once daily, beginning on Day 1 (first prescription day), at least 1 h before dinner for 7 days.

### 2.4 Endpoints

The primary endpoint was the average value of the total symptom score (TSS) at Day 5-7. An important secondary endpoint was the change of quality of life measures, in terms of the Japanese version of the Epworth Sleepiness Scale (JESS) from baseline at Week 1 after intervention. Other secondary endpoints included Urticaria activity score (UAS) 7 (The sum of the daily UAS scores over 7 consecutive days), change from baseline in total DLQI score at Week 1 after the intervention, and average TSS from 3 days before the intervention to the average TSS at Days 5–7 after starting the intervention (22). Adverse event was defined as any disease, disability, death, or infection that occurs during this study. Adverse events and adverse drug reactions were recorded to assess the safety endpoint.

### 2.5 Statistical analysis of the endpoints

Statistical analyses were performed using the program SAS (Statistical Analysis Software 9.4, SAS Institute Inc, Cary, North Carolina, USA). As demonstrated in our protocol study (22), full data sets obtained from all registered participants who were administered at least one medication were used for all analyses. Detailed analyses of endpoints are presented in the published protocol (22). The difference between the groups was estimated based on an Analysis of covariance (ANCOVA) model with the group and stratification factor UCT (<8 points and ≥8 points) as covariates. The adjusted difference in means between both groups with the ANCOVA and its 95% confidence interval was also estimated. According to a domestic phase III randomized controlled trial conducted for the approval of bilastine in treating CSU (18), the mean difference between the bilastine and placebo groups was approximately 1.7. Based on this observed difference, the non-inferiority margin was set at 0.8, which is less than half of the mean difference. The significant secondary endpoints was examined for the superiority of the bilastine switching group. The significance level was considered at one-sided p- value of 0.025 (22). To adjust for multiplicity of testing, based on the closed procedure, if the primary analysis for the primary endpoint showed statistical significance, we proceeded to compare the superiority between the two groups for JESS; if the primary analysis for the primary endpoint shows a statistical significance, the superiority between the two groups were compared (22). As a *post-hoc* analysis, we compared the effects of oral bilastine according to the type of H1AH the patients had taken before participating in the trial.

### 2.6 Ethical approval and informed consent

This study was conducted in accordance with the Clinical Trials Act; the study protocol complied with the Declaration of Helsinki, was approved by Kobe University Clinical Research Ethical Committee, and registered in the Japan Registry of Clinical Trials (Identifier: jRCTs051180105) in accordance with recommendations of the International Committee of Medical Journal Editors (Registered on March 8, 2019; https://jrct.niph.go.jp/latest-detail/jRCTs051180105). Ethics review and approval was conducted at all participating facilities except for the main facility, Kobe University. Written informed consent was obtained from all participants.

## 3 Results

### 3.1 Participant selection, baseline demographics, and clinical characteristics

**Figure 1:**
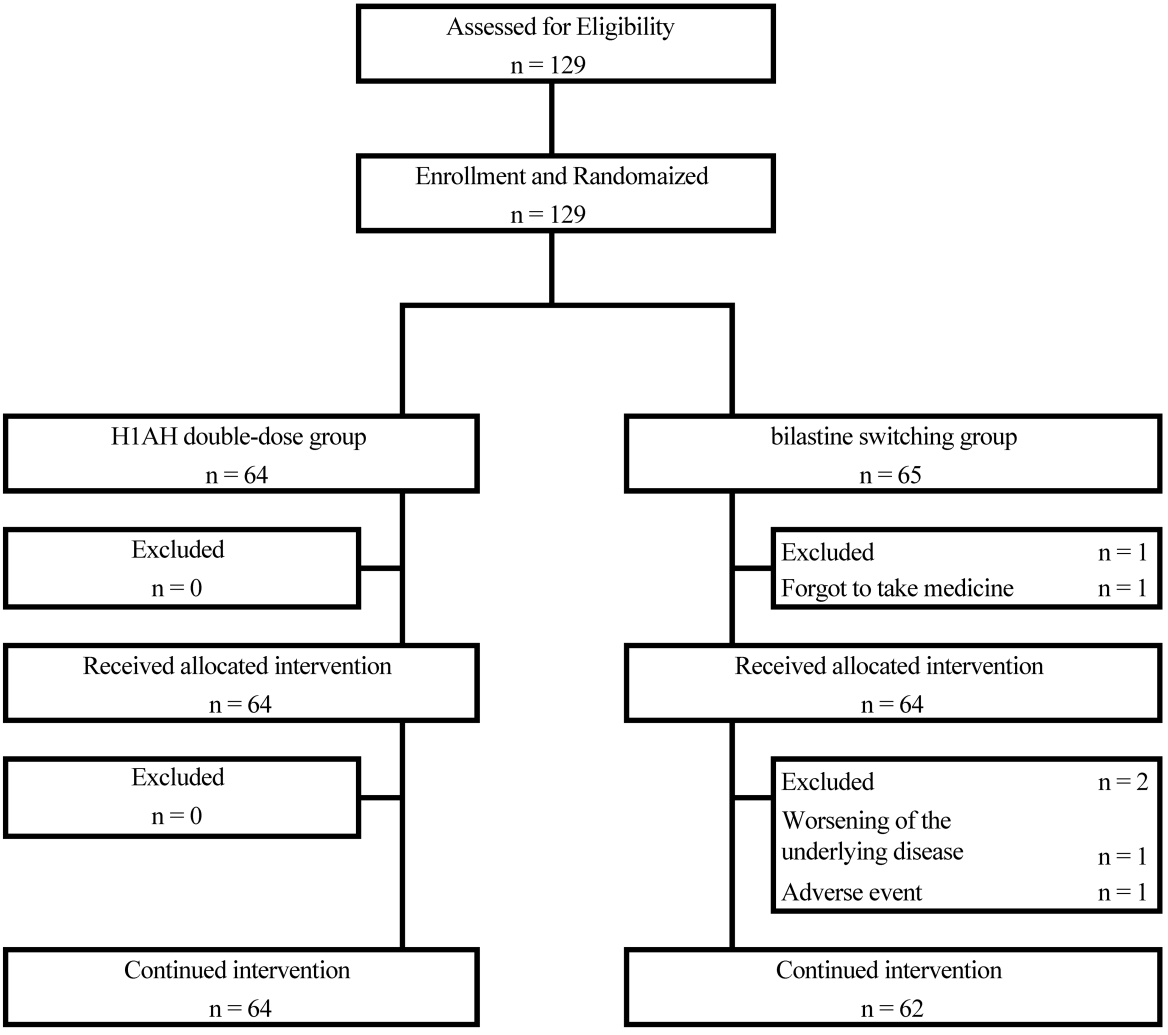
Patient flow.

The participant selection flow is shown in **Figure 1**. Of the 129 patients, 64 were randomized to the H1AH double-dose group and 65 to the bilastine-switching group. One patient in the bilastine-switching group did not start study treatment because he missed a dose on the first day of study drug administration. With the exception of this one patient, 128 patients were started on study treatment, 64 in the H1AH double-dose group and 64 in the bilastine-switching group, all of whom had FAS and SAS (**Figure 1**).

The patients’ baseline demographics and clinical characteristics are summarized in **Table 1**.

**Table 1:** Baseline demographic and clinical characteristics.

Characteristics		H1AH double-dose group (n=64)	Bilastine switching group (n=64)
Age	Mean(SD)	52.3(18.4)	49.4(18.0)
Sex	Male, n(%)	12(18.8%)	22(34.4%)
	Female, n(%)	52(81.3%)	42(65.6%)
UCT (category)	< 8, n(%)	39(60.9%)	39(60.9%)
	8 ≤, n(%)	25(39.1%)	25(39.1%)
UCT (score)	Mean(SD)	6.5(2.6)	6.7(2.4)
Months after first onset of urticaria	Median(range)	77.6(1.5, 674.6)	69.9(1.5, 620.4)
JESS (DAY1)	Mean(SD)	8.3(5.0)	6.8(5.1)
DLQI (DAY1)	Mean(SD)	6.3(5.1)	5.2(4.9)
Complication	No, n(%)	30(46.9%)	23(35.9%)
	Yes, n(%)	34(53.1%)	41(64.1%)
	Physical urticaria, n(%)	6(9.4%)	5(7.8%)
	Angioedema, n(%)	8(12.5%)	4(6.3%)
	Autoimmune disease, n(%)	6(9.4%)	3(4.7%)
	Liver dysfunction, n(%)	2(3.1%)	2(3.1%)
	Renal dysfunction, n(%)	0(0%)	0(0%)
	Food allergy, n(%)	4(6.3%)	2(3.1%)
	Atopic dermatitis, n(%)	1(1.6%)	1(1.6%)
	Allergic rhinitis, n(%)	7(10.9%)	10(15.6%)
	Asthma, n(%)	4(6.3%)	2(3.1%)
	Others, n(%)	20(31.3%)	21(32.8%)
Pretreatment medication before enrollment	Tricyclic drugs	26	21
	Olopatadine hydrochloride	11	10
	Loratadine	3	1
	Epinastine hydrochloride	3	3
	Lupatadine fumarate	9	6
	Desloratadine	0	1
	Piperazine Derivatives	38	43
	Fexofenadine	10	13
	Levocetirizine	9	7
	Bepotastine besilate	16	20
	Cetirizine	1	2
	Ebastine	2	1
Status of taking medication	80%≤ of Prescribed Dose	64(100%)	62(96.9%)

H1AH, Histamine H1 receptor antagonist; UCT, Urticaria control test; n, number; SD, standard deviation.

### 3.2 Primary endpoint

The difference in TSS between the bilastine switching group and the H1AH double-dose group was 0.17 (95% CI -0.32, 0.67), which was smaller than the non-inferiority margin of 0.8, and the p-value of the test was 0.007 (**Supplementary Figure 1**).

**Figure 2:**
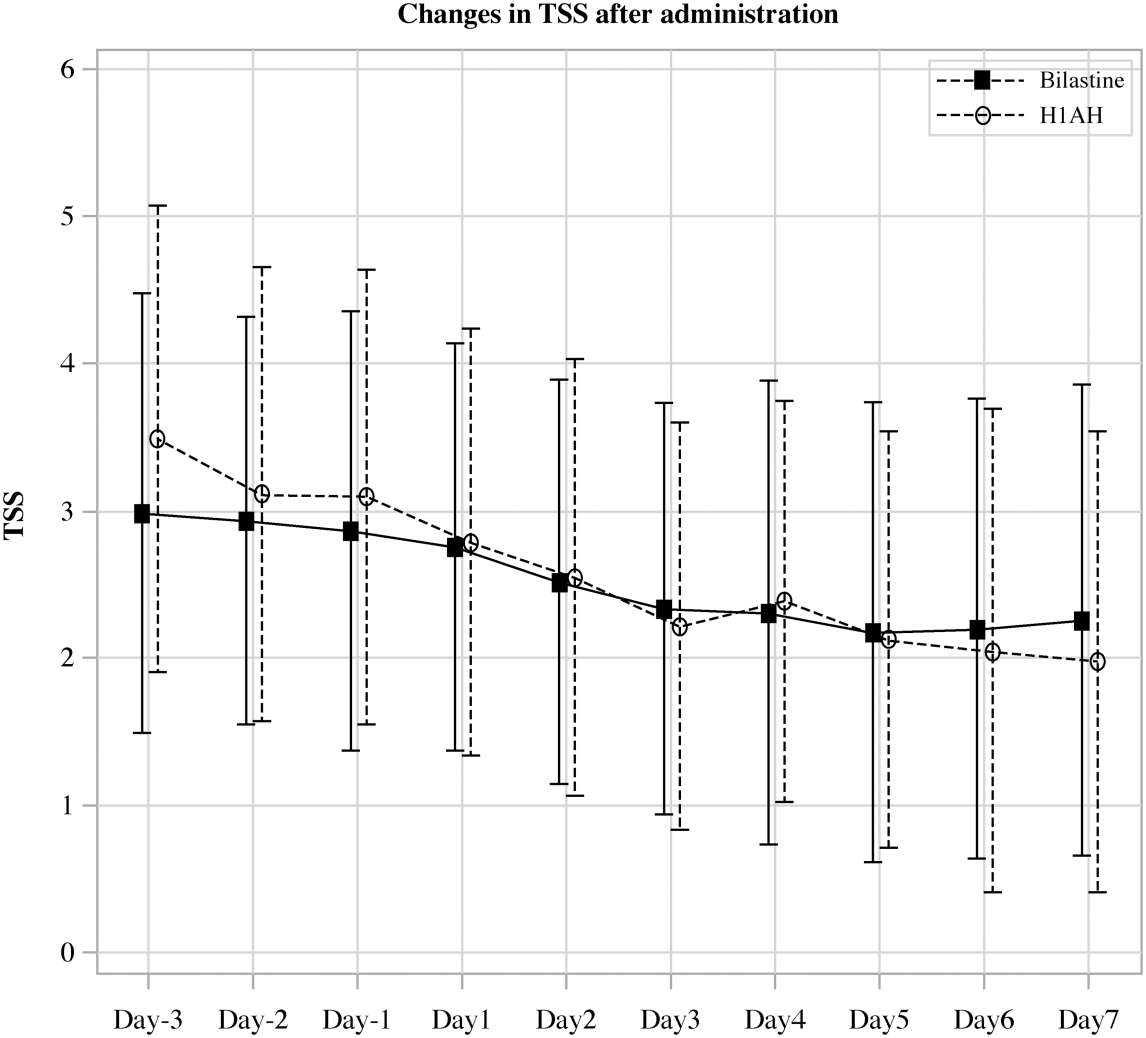
Change in the average value of daily total symptoms score in the H1AH double-dose and bilastine switch groups after start of treatment.

Therefore, the null hypothesis “difference ≥ 0.8 between the means of the bilastine switching group and the H1AH doubling group in the population” was rejected, indicating non-inferiority of the bilastine switching group to the H1AH doubling group for the mean value of TSS 5 to 7 days after the start of treatment (**Table 2**; **Figure 2**). The sensitivity analysis yielded results that were consistent with those of the primary analysis, indicating robustness in the findings (**Supplementary Table 3**). When stratified in terms of UCT (<8 and ≥8), the difference in TSS between the groups was smaller in patients with high UCT (0.01 (95% CI -0.62, 0.64)) than those with low UCT (0.03 (95% CI -0.43, 1.02)) (**Supplementary Figure 1**). TSS 5 to 7 days after the start of treatment became 0 in 7/62 (11.3%) of the bilastine switching group and 5/64 (7.8%) of the H1AH double-dose group. There was a gender imbalance between the groups: The H1AH doubling group included 18.8% men, while the bilastine switching group included 34.4% men. We conducted a subgroup analysis to examine the interaction between treatment effect and gender. The p-value for interaction was 0.77, and there was no significant difference in treatment effect between the subgroups based on gender.

**Table 2:** The difference in primary endpoint and secondary endpoints between groups.

Endpoint	H1AH double-dose group (n=64) mean (SD)	Bilastine switching group (TSS, DLQI, n=62; JESS, UAS7, n=63) mean (SD)	Difference between group (95%ci) mean (SD)	p value
Primary Endpoint				
Total Symptoms Score^‡^	2.04(1.44)	2.21(1.48)	0.17(-0.32, 0.67)	0.007^*^
UCT<8 UCT≥8	2.31(1.57)	2.61(1.58)	0.30 (-0.43, 1.02)	
	1.64(1.12)	1.63(1.09)	0.01 (-0.62, 0.64)	
Secondary Endpoint				
JESS (change from baseline)^§^	-0.3 (3.8)	-0.4 (2.9)	-0.07 (-1.26, 1.11)	0.4516^†^
UCT<8	-1.2 (3.6)	-0.4 (3)	0.77(-0.75, 2.30)	
UCT≥8	1 (3.7)	-0.3 (2.9)	-1.36 (-3.26, 0.54)	
UAS7^§^	17.3 (8.5)	17.4 (9.1)	0.07 (-2.91, 3.05)	0.5181^†^
UCT<8	19.2 (9.3)	19.5 (9.5)	0.35 (-3.93, 4.62)	
UCT≥8	14.4 (6.3)	14.1 (7.4)	-0.36 (-4.27, 3.55)	
DLQI (change from baseline)^‡^	-2.5 (3.8)	-2.5 (3.9)	0.02(-1.29, 1.30)	0.9794^†^
UCT<8	-3.6 (4.2)	-3.3 (4.3)	0.34 (-1.60, 2.29)	
UCT≥8	-0.7(2.0)	-1.2 (2.7)	-0.48 (-1.85, 0.89)	

^*^p-value (one-sided) based on test of non-inferiority with margin 0.8.

^†^p-value for superior test (null hypothesis ≧ is 0).

^‡^Data on Total Symptoms Score and DLQI were available for 62 cases in Bilastine switching group due to omissions in filling out the questionnaire.

^§^Data on JESS and UAS7 were available for 63 cases in Bilastine switching group due to omissions in filling out the questionnaire.

UCT<8 and UCT≥8 indicate the values for each group when stratified.

TSS, Total symptoms score; JESS, Japanese version of Epworth sleepiness scale; UAS7, Urticaria activity score over 7 consecutive days; DLQI, Dermatology life quality index.

### 3.3 Important secondary endpoints

Since non-inferiority was demonstrated for the primary endpoint of mean TSS at 5 to 7 days after the start of treatment, a test for the change in JESS at 1 week after the start of treatment was performed. The between-group difference in change in JESS after 1 week of treatment was -0.07 (95% CI -1.26, 1.11). The null hypothesis was “the difference between the means of bilastine switching group and H1AH double-dose group in the population ≥ 0.” The p-value of the test of means was 0.4516, and the null hypothesis was not rejected. These results did not indicate any superiority of the bilastine-switching group over the H1AH double-dose group for sleepiness evaluated using JESS (**Table 2**). When stratified in terms of UCT (<8 and ≥8), the numerical reduction in JESS in the bilastine switching group was more prominent when UCT≥8 (-1.36 (95% CI -3.26, 0.54)) (**Table 2**), indicating that compared with the control group, switching to bilastine may be more beneficial regarding sleepiness.

### 3.4 Secondary endpoints

For the secondary endpoint, there was no difference between the bilastine switching group and the H1AH double-dose group for UAS7 (0.07 (95% CI -2.91, 3.05); p=0.5181) and DLQI (0.02 (95% CI -1.29, 1.30); p=0.9794) (**Table 2**). The results of subpopulation analysis by UCT for each evaluation item are listed in **Table 2**. No statistically significant differences in UAS7 and DLQI were observed between H1AH double-dose group and bilastine switching group.

### 3.5 Safety evaluations

No severe adverse events occurred. In the H1AH double-dose group, 9 out of 9 adverse events occurred in 5 (7.8%) subjects and in the bilastine switching group, and five of the adverse events occurred in 3 (4.7%) subjects. Because somnolence, malaise, dry mouth, headache, and dizziness were considered to be possible H1AH-related adverse event (H1AH-related illnesses), H1AH-related adverse events occurred in 5 cases (8 items) in the H1AH double-dose group and 2 cases (3 items) in the bilastine switching group (**Table 3**).

**Table 3:** Adverse events observed in two groups.

	H1AH double-dose group n=64	Bilastine switching group n=64
	Cases (%)	Cases (%)
At least one Adverse Event, n(%)	5 (7.8)	3 (4.7)
Count of adverse event, n	9	5
Atopic dermatitis, n (%)	1 (1.6)	0 (0.0)
Nausea, n (%)	0 (0.0)	1 (1.6)
Somnolence, n (%)	4 (6.3)	1 (1.6)
Malaise, n (%)	2 (3.1)	0 (0.0)
Dry mouth, n (%)	1 (1.6)	0 (0.0)
Headache, n (%)	1 (1.6)	1 (1.6)
Dizziness, n (%)	0 (0.0)	1 (1.6)
Urticaria, n (%)	0 (0.0)	1 (1.6)

Underline: H1AH-related adverse event.

### 3.6 *Post-hoc* analysis

Subjects who were taking tricyclic H1AH (mean TSS; 2.7) before switching to bilastine showed higher mean TSS on 5 to 7 days after starting treatment than those who were taking piperazine/piperidine H1AH (mean TSS; 2.0) (**Table 4**).

**Table 4:** TSS corresponding to different types of drugs taken before registration in the bilastine group.

	Medication before enrollment		Difference between groups (SD)	p value
	Tricyclic drugs group n=21	Piperazine/Piperidine drugs n=41		
Total Symptoms Score	2.7 (1.7)	2.0 (1.3)	-0.780 (1.44)	0.0475

## 4 Discussion

To the best of our knowledge, our clinical trial is the first well-conducted study offering strong evidence of non-inferiority and safety of switching H1AH treatment when compared with double-dose H1AH treatment in CSU patients refractory to standard dose H1AH in the Japanese population. The efficacy and safety of switching to bilastine were evaluated in comparison to doubling-up the standard dose of H1AH (non-sedating sgAH). For CSU, H1AHs (non-sedating sgAH) are the first-choice drug recommended in both international and Japanese guidelines. However, only less than 50% of patients with CSU achieve absence of symptoms by H1AH treatment (9). For patients with refractory CSU, the recommended step one of treatment algorithm in these guidelines is increasing the dose of antihistamines (23, 24). In contrast to the international scenario, the health insurance system in Japan limits the dose increase of H1AH to two times. Thus, doubling up the standard dose as well as switching to other H1AH are recommended in the Japanese guideline. Therefore, treating Japanese patients with refractory CSU, switching to another H1AH is considered an alternative treatment strategy for refractory CSU.

In this multicenter, open-label, randomized, parallel-group comparative study (H1-SWITCH), we divided patients with CSU who were refractory to H1AH other than bilastine into a doubling-dose group and a switching group to bilastine allocating 64 patients in each group. Reflecting the real-world medical practice for CSU patients in Japan, we limited the increase in H1AH dose to twice the standard dose (25). We set TSS as the primary endpoint and the JESS as the important secondary endpoint. We designed the trial for the non-inferiority of bilastine compared with other H1AHs for TSS and the superiority of bilastine compared with other H1AHs for JESS. To confirm the superiority for the important secondary endpoint, the statistical testing for JESS was limited to only when the non-inferiority for the primary endpoint, TSS, was shown. This allowed us to avoid type I error inflation (22). The observed difference was smaller than 0.8- the pre-specified non-inferiority margin, indicating non-inferiority of the bilastine switching group to the H1AH double dose group in terms of TSS values 5 to 7 days after administration. Furthermore, it was found that the actual TSS values tended to decrease before and after the intervention of switching to bilastine or doubling the dose (**Figure 2**). Regarding JESS, although we did not demonstrate statistical superiority, the scores were almost similar in both groups, suggesting comparable effectiveness between the treatments.

In this study, we observed a gender difference between the groups (male:18.8% vs. 34.4%), and there was no difference between subgroups based on sex in TSS. Few studies have mentioned gender differences in the efficacy of antihistamines. Hide et al. conducted a subgroup analysis on the efficacy of bilastine by gender and reported no significant differences (18). Based on this, we believe that the impact of the gender imbalance on the results is minimal, if any.

Bilastine, the key drug in this study, is a non-sedating piperazine derivatives sgAH that is approved for the treatment of urticaria and allergic rhinitis in over 90 countries. In Japan, efficacy and safety have been proven in a double-blind, placebo-controlled, randomized phase II/III study prior to insurance approval for CSU (18), and in a one-year post-marketing survey for CSU (17). A clinical pharmacological study using positron emission tomography demonstrated that a single oral dose of bilastine 20 mg did not occupy H1 receptors in the brain. Based on this, bilastine 20 mg has been approved for insurance coverage in Japan. Existing reports suggested ethnic differences in prevalence of chronic urticaria between Asian and Western populations (1, 26–28). Systematic review of clinical studies from 16 countries showed that angioedema was more prevalent in European and American patients with CSU than their Asian counterpart and treatment escalation from first generation and second generation H1-antihistamines and treatment changes were common in a majority of patients (29). The real-world experiences by the panel members of the Original Real-world cases of Bilastine In Treatment (ORBIT) study with ‘difficult- to treat’ cases of CSU from Southeast Asia region showed that once-daily use of bilastine 10 mg (children) and 20 mg (adults/adolescents) was well tolerated and effective in long-term management of CSU and inducible urticaria (30). In these cases of refractory CSUs treatment decision involving bilastine was driven by its non-sedating nature and cost-effectiveness and an impressive safety or tolerability profile with no immunosuppressive effects. In addition to considering racial differences in the symptoms and management of CSU, our study offers two advantages to Japanese patients with CSU: non-sedation and cost-effectiveness.

In Japan, the two-stage treatment goal of urticaria is to achieve 1) a symptom-free condition by continuous use of medications, and 2) to achieve both symptom and drug-free condition. In 2018, the JDA published the updated treatment guidelines for CSU (25). It provided a treatment algorithm for urticaria treatment in the Japanese population as follows; the first line of treatment includes non-sedating second-generation H1AH (including doubling up the standard dose, switching, and combination of the two H1AH). Previously, in a randomized, placebo-controlled trial of adult Japanese patients with CSU, compared to placebo, the use of bilastine (20 or 10 mg) once daily significantly improved the primary efficacy outcome, in terms of change in TSS from baseline (18). Retrospective analysis of data of patients with chronic urticaria showed switching from the current sgAH to another sgAH benefitted 14.8% of patients in terms of remission with a standard dose of sgAHs (31). In the Asian context of CSU management, however, a few retrospective studies have shown promising results. One study showed that Indian patients who responded inadequately to a double dose or combined use of commonly used antihistamines, achieved relief from symptoms of CSU and improved the quality of life after switching over to bilastine (20). In another study, in patients with inadequate response, switching over to bilastine from a standard dose of commonly used antihistamines resulted in improved CSU symptom management and satisfaction with the drug (32). However, there is a dearth of randomized clinical trials that have assessed the efficacy of switching to bilastine in patients with CSU who were refractory to standard dose H1AH. The findings from our randomized controlled trial provides the first high-quality evidence of benefit of switching to bilastine (20mg) showed non-inferior efficacy to double-dose H1AH among Japanese patients. Since 10 mg of bilastine has been shown to be as effective as 20 mg of bilastine in CSU (18), we speculate that 20 mg of bilastine was comparable to other double-dose H1AH in our study.

Some additional aspects of treatment-related insights emerged from the present trial. When comparing UCT, which is a stratification factor, we observed a tendency for TSS to decrease better at H1AH double dose group in those with UCT points less than 8 points, which reflect severe symptoms with poor control of symptoms, but not 8 points or more (**Table 2**). This suggests that in patients with poorer control, there may be benefit from doubling the dose compared to switching. When the two groups were compared on UAS7 reflecting disease activity during 7 consecutive days, a positive urticaria severity score, no difference was found between the bilastine switch and the H1AH double dose groups. This is interpreted as switching to bilastine leads to outcome non-inferior to H1AH double-dose therapy in H1AH-resistant CSU even when symptom indicators other than TSS are used.

Next, regarding the change in QoL, a secondary endpoint, although no difference between the bilastine switching group and the H1AH double dose group was observed, a decrease in DLQI of approximately 2.5 from the baseline was observed in each group within one week of administration. (**Table 2**). When health related QoL was assessed in terms of DLQI, bilastine 20 mg showed efficacy similar to levocetirizine 5 mg an extensively investigated H1AH in improving the QoL by reducing the general discomfort and disruption of sleep associated with CSU (28). In a retrospective study involving patients with CSU, at week 24 DLQI improved significantly from baseline by using bilastine (20).

In Japanese patients with CSU, long-term treatment with bilastine 20 mg once daily for 52 weeks was reported as safe and well tolerated (18). In our previous randomized phase II/III, double blind study among Japanese patients with CSU, similar types of mild or moderate intensity AEs occurred across placebo, 10 mg, and 20 mg bilastine (once daily for two weeks) groups. During follow up at 4-7 days of treatment completion AEs related to nervous system disorders in terms of somnolence and headache were reported in 2.0% of patients receiving bilastine 20 mg and 3.0% patients receiving in bilastine 10 mg; and dizziness and hypesthesia in 1.0% (1/100, each) in bilastine 10 mg (18) and these incidences were comparable to the placebo group. In another study, somnolence was reported lesser in patients receiving bilastine 20 mg (5.8%) as compared to levocetirizine 5 mg (6.7%) (27). In the present clinical trial, there was no case of serious adverse events owing to this side effect. In order to verify the superiority of the bilastine switching group in terms of sleepiness, we compared the amount of change in JESS, which was set as an important secondary endpoint, one week after the start of administration, but no superiority of the bilastine switching group was demonstrated over the H1AH double dose group (**Table 2**). This may be due to the fact that the JESS score at the time of intervention was used to evaluate conditions such as sleep apnea, and as it is an evaluation system that easily detects relatively strong sleepiness, it was difficult to detect changes. In fact, at baseline, the JESS score was 8.3 in the H1AH group and 6.8 in the bilastine switching group, which was considered a mild level of sleepiness (**Table 1**). It is unclear if JESS is an appropriate measure of sleepiness in H1AH. A more H1AH-specific measure of quality of life may be needed. To note, the bilastin switching group tended to have fewer cases of somnolence and fewer overall adverse events related to increased dose of H1AH. When stratified in terms of UCT, the numerical reduction in JESS in bilastine switching group was more prominent in cases with better control of symptoms (UCT≥8) (**Table 2**). This observation suggests that switching to bilastine, a less sedating alternative, may have more safety benefits in moderately controlled patients. H1AH-related side effects such as somnolence and fatigue occurred only as 8 incidences in 5 cases (7.8%) in the double-dose group, and 4 incidences in 2 cases (3.1%) in the bilastine switching group (**Table 3**). This finding corroborated with our previous study that reported somnolence related to bilastine was reported in only two of 197 CSU patients (1.0%) (18), and it was considerably lower than that reported in other second-generation H1AH clinical studies (19). The experience from this trial underscores that while prescribing H1AH therapy clinicians should consider all potential adverse drug reactions and the corresponding management strategy should be provided as a guideline.

This study had several limitations. First, the results of this study did not reach the pre-specified sample size of cases. The planned number of patients enrolled was 150 (75 in the H1AH double-dose group, 75 in the bilastine switching group), but due to the COVID-19 pandemic occurring during the study period, only 129 were enrolled during the enrollment period. Second, the study was an open study and was not blinded, which may affect the objectivity of the results, especially subjective assessments such as the patient-reported outcomes. Third, the study was short in duration, and the future course of urticaria symptoms was unknown. Fourth, this study compared double-dose H1-AH dose with bilastine switch, and did not compare it with increasing to 4-fold as recommended in the international guidelines (8), and therefore it is difficult to generalize in the international guidelines.

This was the first randomized controlled trial of the efficacy and safety of switching to bilastine versus double-dose H1AH in patients with CSU who had persistent symptoms after receiving regular doses of 2^nd^ generation H1AH other than bilastine. In terms of efficacy, non-inferiority was demonstrated. Since non-inferiority was demonstrated in terms of efficacy, and there were no severe adverse reactions between the two groups in terms of safety, our results show that switching treatment to bilastine has the same efficacy and safety as treatment with a double- dose of H1AH. Further studies are warranted for evaluating long-term clinical outcomes.

## Data Availability

The original contributions presented in the study are included in the article/**Supplementary Material**. Further inquiries can be directed to the corresponding author.
