# Supplementary material for: Efficacy and safety of switching to bilastine, an H1-antihistamine, in patients with refractory chronic spontaneous urticaria (H1-SWITCH): a multicenter, open-label, randomized, parallel-group comparative study
Source: Front Immunol. 2024 Sep 16;15:1441478. doi: 10.3389/fimmu.2024.1441478 (PMC11439774; doi:10.3389/fimmu.2024.1441478)
Supplement: Supplementary file 1 [file DataSheet1.docx]

Supplementary Material

# Supplementary Figure and Tables

## Supplementary Figure


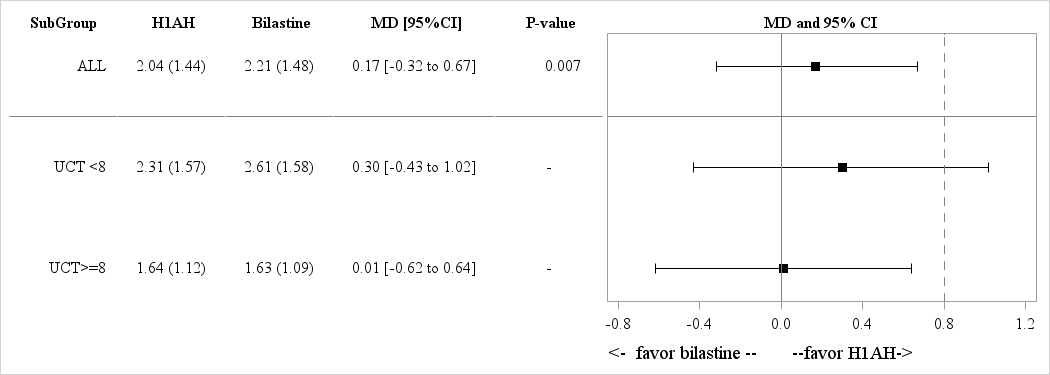


**Supplementary Figure 1.** Forest plot of the difference in TSS between the bilastine switching group and the H1AH double-dose group. MD: mean difference; CI: confidence interval; dash line: non-inferiority margin

## Supplementary Tables

**Supplementary Table 1**. Summary of study assessments and procedures

|  | | | Screening | Test drug/Control drug treatment period | | | | | | | | Withdrawal　(Visit) |
| --- | --- | --- | --- | --- | --- | --- | --- | --- | --- | --- | --- | --- |
|  |  |  |  | Day1  (Visit)  ^※1^ | Day2 | Day3 | Day4 | Day5 | Day6 | Day7 | Day8  (Visit) |  |
| Informed consent | | | ● |  |  |  |  |  |  |  |  |  |
| Participant Background | | | ● |  |  |  |  |  |  |  |  |  |
| Anamnesis & Complications | | | ● |  |  |  |  |  |  |  |  |  |
| Present Medical Condition | | | ● |  |  |  |  |  |  |  |  |  |
| UCT | | | ● |  |  |  |  |  |  |  |  |  |
| Randomization | | |  | ● |  |  |  |  |  |  |  |  |
| Patient diary | | |  | ● ^※2^ |  |  |  |  |  |  | ● ^※2^ | ● ^※2^ |
| TSS | rash | |  | ● ○^※3,4^ | ○ | ○ | ○ | ○ | ○ | ○ |  | ○ |
|  | pruritus | day  time |  | ● ○^※3,4^ | ○ | ○ | ○ | ○ | ○ | ○ |  | ○ |
|  |  | nighttime |  | ● ○^※3,4^ | ○ | ○ | ○ | ○ | ○ | ○ |  | ○ |
| UAS | | |  | ○ | ○ | ○ | ○ | ○ | ○ | ○ |  | ○ |
| JESS | | |  | ● ^※3^ |  |  |  |  |  |  | ● | ● |
| DLQI | | |  | ● ^※3^ |  |  |  |  |  |  | ● | ● |
| Adverse events | | |  |  |  |  |  |  |  |  |  |  |
| Treatment compliance | | |  | ○ | ○ | ○ | ○ | ○ | ○ | ○ | ● ^※5^ | ○ |

●, Performed by a physician at a medical institution

○, Study subjects to perform at home

※1, Screening and Day 1 were performed on the same day.

※2, Patient diary was delivered on Day 1 and collected on Day 8 or upon withdrawal from the study.

※ 3, TSS, JESSS, and DLQI were administered before taking the test drug or control drug on Day 1.

※ 4, For TSS, the situation in the past 3 days before the start of treatment were investigated.

※ 5, The morning oral condition was confirmed on day 8.

**Supplementary Table 2.** Inclusion criteria

| (1) Patients aged 20 years or older at the time that they provide consent |
| --- |
| (2) Patients diagnosed with CSU, which is characterized by the spontaneous appearance of wheals, angioedema, or both for >6 weeks without any triggers, for which second-generation non-sedative or mild-sedative antihistamines (regular dose) is not sufficiently effective at the time of randomization |
| (3) Patients with CSU with pruritus or wheal continuation despite continued treatment with second-generation non-sedating or mild-sedative antihistamines (regular dose) for more than 1 week before consent acquisition. However, it is permissible to change the type of antihistamine within the range of dosage and usage information on the medication information leaflet. Second-generation non-sedating antihistamines include fexofenadine hydrochloride, levocetirizine, olopatadine hydrochloride, bepotastine besylate, loratadine, cetirizine hydrochloride, epinastine hydrochloride, ebastine, lupantadine fumarate, azelastine hydrochloride, and mequitazine. |
| (4) Patients with a urticaria control test (UCT) score of 11 or less on the registration date (UCT can evaluate retrospectively the level of urticaria control over the past 4 weeks using four questionnaires with a recommended cutoff value of 12 for a controlled disease.) |
| (5) Patients for whom documented consent has been obtained regarding their voluntary participation in this clinical study |
| (6) Patients who are able to take the test drug or control drug for 7 days even if symptoms (pruritus or wheal) improve within this period. |
|  |
| Exclusion criteria |
| (1) Patients with urticaria, other than CSU, with an identifiable trigger/cause. If triggering factors are specified so that the symptoms developed in response to this factor can be clearly distinguished from those of CSU, they are not considered to conflict with the exclusion criteria. |
| (2) Patients with a skin disease accompanied by chronic pruritus other than CSU (eczema, contact dermatitis, and atopic dermatitis) |
| (3) Patients with hypersensitivity to bilastine |
| (4) Patients with chronic, uncontrolled medical condition(s) that may increase the risk of study subjects by participating in this clinical study, based on the study investigator’s or study team physician’s judgement. |
| (5) Pregnant or lactating women |
| (6) Patients treated with bilastine, adrenocorticosteroid, or cyclosporine within 4 weeks before obtaining consent. Patients treated with first-generation antihistamines, H2 receptor antagonists, or anti-leukotriene drugs within 1 week before obtaining consent. However, any use of external medicines is permitted. |
| (7) Patients with CSU treated with omalizumab in the past. |
| (8) Patients who are judged as inappropriate by a study investigator or sub-investigators. |

**Supplementary Table 3.** Sensitivity analysis for the Primary Endpoint

| **Endpoint** | Difference between | *p value |
| --- | --- | --- |
|  | groups (95%CI) |  |
| Primary Endpoint |  |  |
| Total Symptoms Score | 0.32(-0.11, 0.75) | 0.0141 |
| UCT＜8 | 0.49 (-0.09, 1.08) | 0.1491 |
| UCT≥8 | 0.05 (-0.57, 0.66) | 0.0085 |

Difference between groups was adjusted by baseline TSS

*One-sided p-value based on a test of non-inferiority with a margin of 0.8.

**Supplementary Table4.** H1RH medications taken by patients prior to study enrollment in two groups

|  | H1AH double-dose group n=64 | | | | Bilastine switching group n=64 | | | | |  |
| --- | --- | --- | --- | --- | --- | --- | --- | --- | --- | --- |
|  | N (%) | | | |  | N (%) | | | |  |
| Fexofenadine hydrochloride | 10 | ( | 15.6% | ) |  | 13 | ( | 20.3% | ) | |
| Levocetirizine hydrochloride | 9 | ( | 14.1% | ) |  | 7 | ( | 10.9% | ) | |
| Olopatadine hydrochloride | 11 | ( | 17.2% | ) |  | 10 | ( | 15.6% | ) | |
| Bepotastine besilate | 16 | ( | 25.0% | ) |  | 20 | ( | 31.3% | ) | |
| Loratadine | 3 | ( | 4.7% | ) |  | 1 | ( | 1.6% | ) | |
| Levocetirizine hydrochloride | 1 | ( | 1.6% | ) |  | 2 | ( | 3.1% | ) | |
| Epinastine hydrochloride | 3 | ( | 4.7% | ) |  | 3 | ( | 4.7% | ) | |
| Ebastine | 2 | ( | 3.1% | ) |  | 1 | ( | 1.6% | ) | |
| Rupatadine fumarate | 9 | ( | 14.1% | ) |  | 6 | ( | 9.4% | ) | |
| Desloratadine | 0 | ( | 0.0% | ) |  | 1 | ( | 1.6% | ) | |
